# Genetic characterization and phylogenetic study of Indonesian indigenous catfish based on mitochondrial cytochrome B gene

**DOI:** 10.14202/vetworld.2020.96-103

**Published:** 2020-01-13

**Authors:** Dorothea Vera Megarani, Herjuno Ari Nugroho, Zahrah Prawita Andarini, Yura Dwi Risa B. R. Surbakti, Rini Widayanti

**Affiliations:** 1 Department of Clinical Pathology, Faculty of Veterinary Medicine, Universitas Gadjah Mada, Yogyakarta, Indonesia; 2 Research Center for Biology, Indonesian Institute of Sciences (LIPI), Cibinong, West Java, Indonesia; 3 Veterinary Medicine Undergraduate Program, Faculty of Veterinary Medicine, Universitas Gadjah Mada, Yogyakarta, Indonesia; 4 Department of Biochemistry and Molecular Biology, Faculty of Veterinary Medicine, Universitas Gadjah Mada, Yogyakarta, Indonesia

**Keywords:** cytochrome B, Indonesian indigenous catfish, mitochondrial DNA, phylogenetic study, *Siluriformes*

## Abstract

**Aims:**

This study aimed to determine the genetic characterization and phylogenetic structure of Indonesian indigenous catfish using cytochrome B (Cyt B) sequences.

**Materials and Methods:**

The genomes of 26 catfishes caught from nine rivers from nine different geographical locations around Indonesia were analyzed. The tissue isolation method was used to isolate the total genome of the fishes. Furthermore, polymerase chain reaction was done to amplify the mtDNA Cyt B using the CytBF and CytBR primers. Following sequencing, the analysis of genetic variation and the phylogenetic relationship was performed using MEGA version X software.

**Results:**

Cyt B gene sequencing attained a total of 1139 nucleotides encrypting 379 amino acids for all samples. The ClustalW alignment program using MEGA X software revealed 395 substituted nucleotides, which then translated into 63 amino acid variation sites among all 26 samples. No amino acids in catfish BB were different compared to catfish PM, MP, and KR2,3. Catfish MS had one modified amino acid; KR1 and KS had two different amino acids; BF had 38 different amino acids; EM had 31 different amino acids; and BSBJ had 26 different amino acids compared to catfish BB. The most significant alteration of amino acids was between catfish EM and BF (49 amino acids).

**Conclusion:**

Indonesian catfish were divided into five clades based on the Cyt B gene. Samples KR and MP (Sumatra); MS and BB (Kalimantan); and PM (Java) were clustered with *Hemibagrus nemurus* and *Hemibagrus wyckioides* (Bagridae family). Samples from Kalimantan (KS) and one sample of KR (KR1) from Sumatra were clustered with *Sperata seenghala* and *Hemibagrus spilopterus* (Bagridae family). Samples from Java (BSBJ) were clustered with *Pseudolais pleurotaenia* (Pangasiidae family). Samples EM (Java) were together with *Mystus cavasius* (Bagridae family). Samples from West Papua were clustered with *Potamosilurus latirostris* (Ariidae family).

## Introduction

Indonesia is a country that features exceptional biodiversity, including a wide variety of fish species. There is an exceedingly large number of indigenous fishes that can be found in Indonesia, one of which is catfish (Siluriformes). This group of fish consists of 106 species, which are grouped into 35 genera and 12 families, including Bagridae, Pangasiidae, Siluridae, Schilbeidae, Akysidae, Parakysidae, Sisoridae, Ariidae, Plotosidae, Loricariidae, Clariidae, and Chacidae. These catfishes live in streams of Sumatra, Java, Kalimantan, Sulawesi, and Papua Island [1]. Due to their identical appearance and close relations, there are difficulties in differentiating their species. Furthermore, local people tend to use the same term for anyone of the catfishes which have acquired the recognition of Indonesian for being a good source of protein and other nutritional values, which is Baung catfish [1,2]. A previous study classified this particular catfish into *Hemibagrus nemurus*, which is in the Bagridae family [3]. Nonetheless, another study of Baung catfish originally from East Java Indonesia allocated this catfish into the Pangasiidae family based on the cytochrome C oxidase subunit 3 (COX-III) gene [4]. Therefore, further studies of the genetic markers of these indigenous catfishes, known as Baung catfish to local people in Indonesia, are needed to identify the catfish species and preserve their genetic resources. Such a genetic study is one of the first steps in the conservation strategy of Indonesian indigenous catfishes, which are presently starting to decrease in the wild [5]. The rapid fishing of native catfish without proper preservation and lack of protection from water contamination have caused a significant threat to their population.

Mitochondrial DNA (mtDNA) is widely used as a useful target for species identification and genetic diversity studies due to several reasons, including the number of mtDNA is higher than the nuclear DNA, and it is only passed down by the mother [6]. Cytochrome B (Cyt B) gene is one of the genes which is coded by mtDNA and plays a significant role in the transfer of electrons in the respiratory tract. Molecular analyses of the animal species using the Cyt B gene have been done in preceding studies, including *Haliaeetus leucogaster* [7,8], *Macrocephalon maleo* [9], *Capra hircus* [10], and *Scomberomorus* sp. [11]. This gene is widely used to examine the relationship of the same genus or family and the phylogenetic studies of cuscus from Maluku (*Spilocuscus maculatus*, *Spilocuscus rufoniger*, *Phalanger orientalis*, and *Phalanger vestitus*) [12]. The encoding gene is used as a genetic marker because the codons are based on position and have more conserve and diverse regions [13]. The Cyt B gene has a moderate evolutionary level and a clear evolution pattern, making it suitable for phylogenetic evolution studies at intraspecific and interspecific levels [14,15]. Another mitochondrial gene that can be used for the barcoding is cytochrome C oxidase subunit 1 (COX-I) gene. In contrast with Cyt B, which is commonly used for determining the phylogenetic relationships within families and genera, the COX-I gene is suitable for identifying individuals belonging to the same species and distinguishing between individuals from different species [16,17]. The mutation rate of the CO1 gene sequence is slow enough so that the sequence is identical in the same species, yet it is fast enough so that it would be different between species [16].

This study aimed to study the Cyt B gene of the indigenous catfish from different parts of Indonesia known as Baung catfish for local people and to determine the diversity as well as the phylogenetic relationship among them using Cyt B as the genetic marker and comparing them with the available sequence records from the GenBank. Moreover, the genetic variability of catfish in Cyt B region was measured to get the DNA barcoding of catfish among different regions from the mtDNA sequences.

## Materials and Methods

### Ethical approval

This study was approved by the Animal Ethics Committee for using Animal and Scientific Procedures in the Faculty of Veterinary Medicine, Universitas Gadjah Mada, Indonesia.

### Sample collections

A total of 26 catfish from different parts of Indonesia known as Baung catfish for local people were collected from nine different streams or rivers from eight provinces on four islands [4]. The origin and the total number of the samples collected are presented in Table-1. To prevent any bias in the genetic analysis, each of the samples was taken individually from its habitat, and they were identified based on their morphological features. Tissue samples were first preserved in the RNAlatter buffer (Qiagen) and then used for the total DNA isolation.

**Table-1:** Samples used in this study.

River	Province	Number of samples	Sample codes
Elo	Magelang, Central Java	2	EM1, EM2 [4]
Progo	Magelang, Central Java	3	PM1, PM2, PM3 [4]
Kampar	Pekanbaru, Riau, Sumatra	3	KR1, KR2, KR3 [4]
Musi	Palembang, South Sumatra	3	MP1, MP2, MP3 [4]
Mahakam	Samarinda, East Kalimantan	3	MS1, MS2, MS3 [4]
Kapuas	Sintang, West Kalimantan	2	KS1, KS2 [4]
Bengawan Solo	Bojonegoro, East Java	3	BSBJ1, BSBJ2, BSBJ3 [4]
Barito	Banjarmasin, South Kalimantan	3	BB1, BB2, BB3
Bomberay	Fakfak, West Papua	4	BF1, BF2, BF3, BF4

### DNA extraction and amplification

gSYNC™ DNA Mini Extraction Kit (Geneaid Biotech Ltd., Taipei, Taiwan) was used for the total DNA isolation from the tissue samples according to the kit’s instruction. Extracted total DNA was stored at −20°C until further examination.

### Primer design

Amplification of the target DNA fragments was done by polymerase chain reaction (PCR) using the pair primer of Cyt B 1242 bp, which was designed with Primer3 output program (http://www-genome.wi.mit.edu/cgi.bin/primr3.cgi/results_from-primer3) created on the mitochondrial genetic sequence data of *H. nemurus* (Access number KJ573466.1) and *Mystus vittatus* (KX177968.1). The pair primer sequence utilized for the Cyt B gene amplification from fish samples was Baung_CytBF5’ CCGCTCTGTCACTTTCTTTT 3’ and Baung_CytBR-5’ GCTCATTTGTGTCCTCCTTT 3’ with the melting temperature of 53.1°C and 53°C, respectively.

### Sequencing and phylogenetic analysis

The sequencing of the purified PCR products was done precisely by 1^st^ Base Sequencing INT (Singapore) and then continued with the analysis of the sequences using the MEGA program version X [18]. DNA forward and reverse sequences of Cyt B gene were aligned with ClustalW. Subsequently, the sequences were first edited, followed by the multiple alignments with the sequence data related to *H. nemurus* and other catfishes around the world recorded in GenBank. Since the Cyt B gene data were not fully presented in the GenBank, the Cyt B target sequence which was 1091 nucleotides in length was then analyzed to determine the genetic relationship of the species using the Kimura 2-parameter method, with 1000 replicates of the bootstrap method. In addition, the phylogenetic analysis based on the Cyt B sequence was performed using neighbor-joining (NJ). The phylogenetic tree of the samples and the other catfish sequences from the GenBank was assembled based on the Cyt B sequences to depict the relationships between the species and the clusters between the individuals.

## Results

### Variation of nucleotides and amino acids sequences

**Figure-1:**
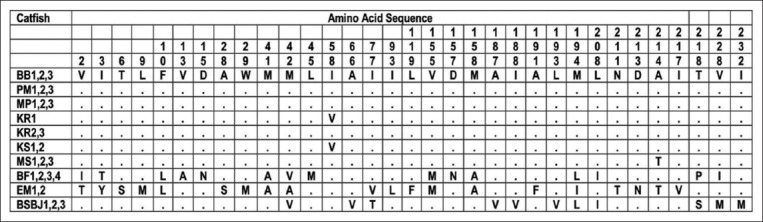
Polymorphic amino acid sites of Catfish from site 2 to 232. Identification with the first sequences is denoted by a dot.

**Figure-2:**
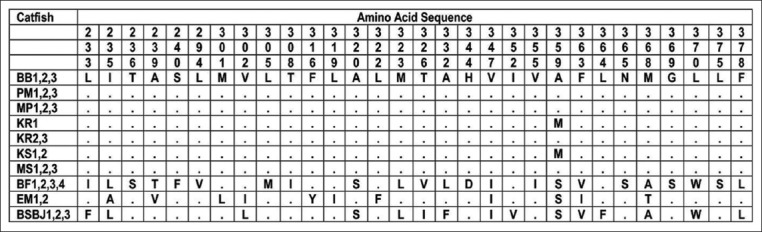
Polymorphic amino acid sites of Catfish from site 233 to 378. Identification with the first sequences is denoted by a dot.

Among the total of 1139 nucleotides Cyt B sequences, which encoded 379 amino acids in 26 samples, there were 395 nucleotides which were substituted. The 395 substituted nucleotides were then translated into 63 amino acid variation sites (Figures-1 and 2). The substitutions of nucleotides resulted in the variation of nucleotides or amino acids [19]. Based on the 63 amino acid variations, there were no amino acids in catfish BB1,2,3 which were different compared to amino acids in catfish PM1,2,3; MP1,2,3; and KR2,3. Nevertheless, catfish MS1,2,3 had one modified amino acid; KR1 and KS1,2 had two different amino acids; BF1,2,3,4 had 38 different amino acids; EM1,2 had 31 different amino acids; and BSBJ1,2,3 had 26 different amino acids compared to catfish BB1,2,3. Further analysis disclosed that the most significant alteration of amino acids was between catfish EM1,2 and catfish BF1,2,3,4, which was 49 amino acids. The amino acid variation of all samples based on Cyt B sequence is presented in Table-2.

**Table-2:** Amino acid variation of Indonesian catfish based on the Cytochrome B gene sequence.

		1	2	3	4	5	6	7	8	9	10
1	BB1,2,3										
2	PM1,2,3	0									
3	MP1,2,3	0	0								
4	KR1	2	2	2							
5	KR2,3	0	0	0	2						
6	KS1,2	2	2	2	0	2					
7	MS1,2,3	1	1	1	3	1	3				
8	BF1,2,3,4	38	38	38	39	38	39	39			
9	EM1,2	31	31	31	32	31	32	30	49		
10	BSBJ1,2,3	26	26	26	27	26	27	27	35	46	

### Phylogenetic relationships between Indonesian catfish and other catfish species

**Figure-3:**
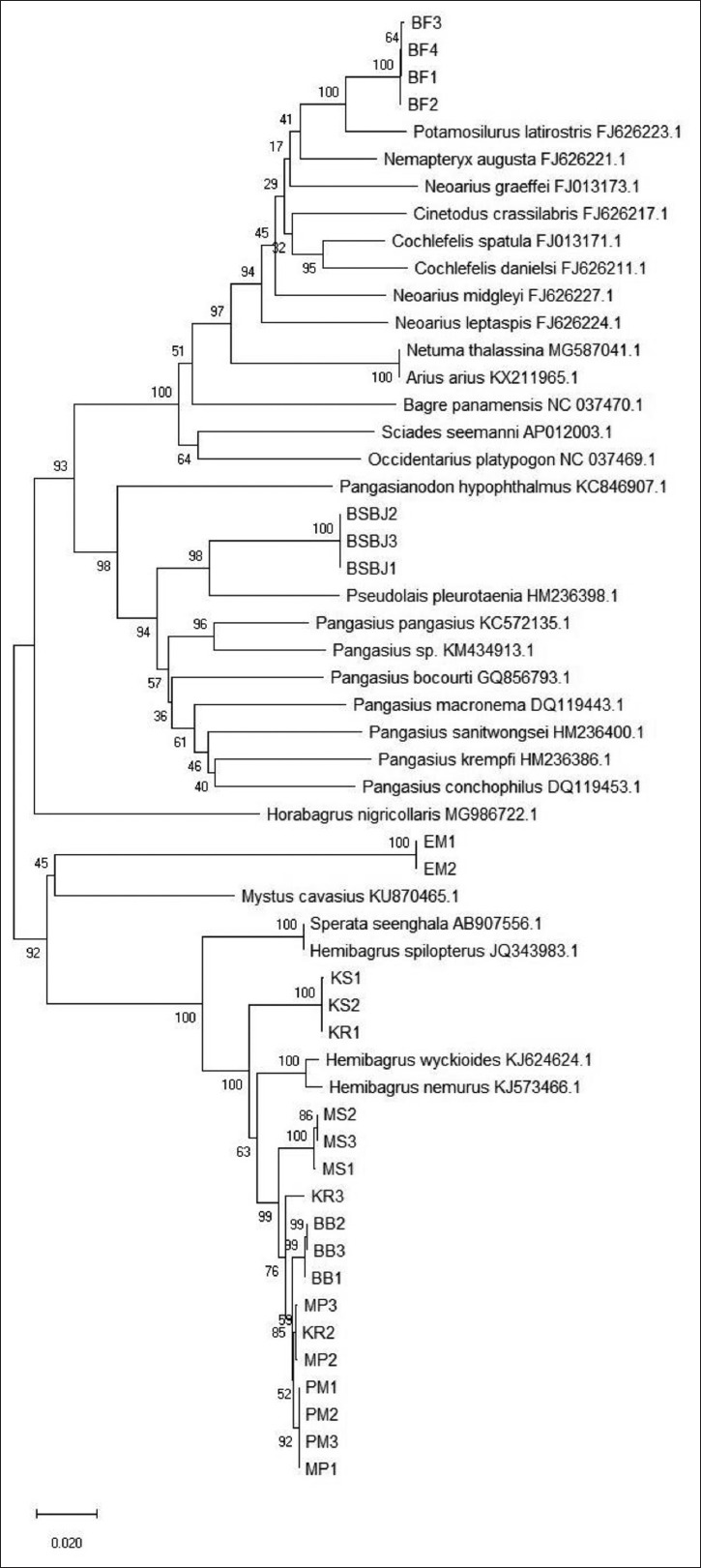
Phylogram of Indonesian catfish based on the cytochrome B nucleotides sequence.

The examination of the phylogenetic relationship of the Indonesian catfish samples and other related catfish species was done starting from the nucleotides site 34^th^-1124^th^ due to the limited data recorded in GenBank. The taxon identification phenogram of the samples was analyzed by constructing the phylogenetic tree using the NJ method. Figure-3 shows the phylogenetic tree of Indonesian catfish based on the Cyt B nucleotides sequence. Figure-3 shows that the Indonesian catfish were separated into five monophyletic groups or clades, in conjunction with the other catfish species around the world. First, Indonesian catfish samples BF1,2,3,4 were clustered with *Potamosilurus latirostris* (FJ626223.1). Furthermore, the samples BSBJ1,2,3 were in the same group with *Pseudolais pleurotaenia* (HM236398.1), while samples EM1,2 were together with *Mystus cavasius* (KU870465.1). Samples KS 1,2 and KR1 were together with *Sperata seenghala* and *Hemibagrus spilopterus*. Finally, samples KR2,3; MS1,2,3; BB1,2,3; MP1,2,3; and PM1,2,3 were in the same cluster with *H. nemurus* (KJ573466.1) and *Hemibagrus wyckioides* (KJ624624.1).

**Figure-4:**
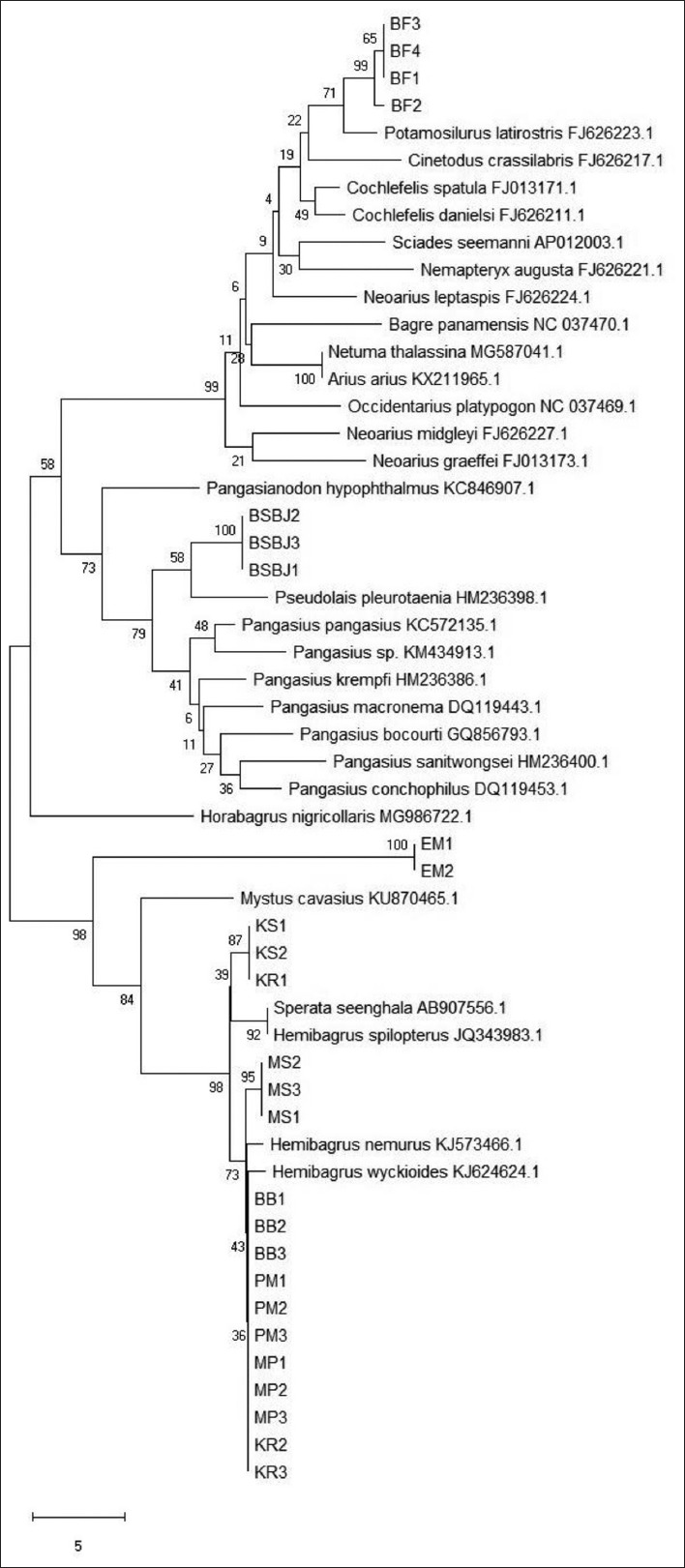
Phylogram of Indonesian catfish based on the cytochrome B amino acid sequence.

The analysis of the phylogenetic tree of Indonesian catfish based on the Cyt B amino acid sequence is shown in Figure-4. Based on the Cyt B amino acid sequence, the Indonesian catfish samples were divided into five different monophyletic groups. Samples BF1,2,3,4 were together with *P. latirostris* (FJ626223.1), whereas samples BSBJ1,2,3 were in the same clade with *P. pleurotaenia* (HM236398.1). In addition, samples EM1,2 were grouped with *M. cavasius* (KU870465.1). The fourth group was formed by samples KS1,2 and KR1, which was in the same clade with *S. seenghala* (AB907556.1) and *H. spilopterus* (JQ343983.1). Finally, the rest of the samples MS1,2,3; BB1,2,3; PM1,2,3; MP1,2,3; and KR2,3 were in the same group with *H. nemurus* (KJ573466.1) and *H. wyckioides* (KJ624624.1).

## Discussion

### Genetic variation of Indonesian catfish based on Cyt B sequence

Catfish (Siluriformes) are frequently found in Asia and Africa throughout fresh-and brackish-water, consisting of more than 200 species in 17 genera and are one of the significant fish orders documented at present [20]. Species catfish from Indonesia which could be found in the rivers of Java, Sumatra, Kalimantan, Sulawesi, and Papua Island, may not possibly be determined based solely on the morphological characteristics. The morphological features are frequently utilized for the identification of other fish species. However, the similarity of morphological features from one catfish species to another can confound the identification of the catfish species [21]. The intention of this research was to determine the molecular characterization and phylogenetic relationship of the indigenous catfish from different parts of Indonesia known as Baung catfish by local people using Cyt B sequence.

In this present research, catfish samples Elo (Magelang, Central Java) and Bengawan Solo (Bojonegoro, East Java) from Java Island had some different amino acids compared to catfish samples from Kalimantan (BB; KS; MS) and Sumatra (KR; MP). These amino acid variations based on Cyt B amino acid sequences supported the confirmation that samples EM and BSBJ were different species than *H. nemurus* based on COX-III sequences [4]. Contrarywise, catfish sample Progo (Magelang, Central Java) which was originally from the different river, yet in the same province and same Java Island with catfish sample from Elo, had the identical amino acids with sample from Barito (Banjarmasin, South Kalimantan), as well as the sample from Musi (Palembang, South Sumatra), and two samples from Kampar (Riau, Sumatera). This investigation indicated that *H. nemurus* exists in Java, Kalimantan, and Sumatera Island. Comparing the catfish samples from Kalimantan (Barito, Mahakam, and Kapuas) and West Papua (Bomberay), there were 38-39 different amino acids between them. The most significant deviation on the amino acids was between catfish samples from Elo (Magelang, Central Java) and catfish samples from Bomberay (Fakfak, West Papua), which had 49 different amino acids. This concluded that the catfish samples from Bomberay were not closely related to other samples from Java, Sumatra, and Kalimantan. In other words, the catfish samples from Papua differed from catfish species from different regions in Indonesia based on the Cyt B amino acid sequence. The analysis of the mitochondrial Cyt B sequence confirmed the similarity of the morphological features of catfish in Indonesia. Therefore, species confirmation using the genetic analysis is truly useful to clarify the taxonomy diversification and evolutionary relationships within this plenteous group of fishes [22,23].

### Phylogenetic and phylogeographic of Indonesian catfish based on Cyt B sequence

*H. nemurus*, initially being from Southeast Asia, had been reported in preceding studies to have an extensive genetic subdivision of the cluster based on the molecular phylogenetics and phylogeography [3,24-27]. To determine the phylogenetic relationship among the catfish from different parts of Indonesia, as well as comparing them with some other catfish species, this present study utilized the genetic sequence records of some species from the Siluriformes order. Constructed by the Cyt B nucleotides sequence, the catfish sample KR and MP (Sumatra); MS and BB (Kalimantan); and PM (Java) were in the same clade with *H. nemurus* and *H. wyckioides*, which was supported by bootstrap 63% NJ. Other catfish samples from Kalimantan (KS) and one sample of KR (KR1) from Sumatra had genetic similarities with *S. seenghala* and *H. spilopterus* (bootstrap 100% NJ). In addition, other catfish samples from Java (BSBJ) were clustered with *P. pleurotaenia* (bootstrap 98% NJ), while samples EM were together with *M. cavasius* (bootstrap 45% NJ). Finally, catfish samples from West Papua formed the same clade with *P. latirostris*, supported by bootstrap 100% NJ.

The phylogenetic tree of Indonesian catfish based on the Cyt B amino acid sequence was identical with the phylogenetic tree based on Cyt B nucleotides, where there were five different clades formed. However, the bootstrap supported that the monophyletic groups were different: Catfish sample KR and MP (Sumatra); MS and BB (Kalimantan); and PM (Java) were together with *H. nemurus* and *H. wyckioides* supported by bootstrap 73% NJ. Catfish samples from Kalimantan (KS) and one sample of KR (KR1) from Sumatra were in the same group with *S. seenghala* and *H. spilopterus* favored by bootstrap 39% NJ. Other catfish samples from Java (BSBJ) were clustered with *P. pleurotaenia* (bootstrap 58% NJ), while samples EM were together with *M. cavasius* (bootstrap 98% NJ). Finally, catfish samples from West Papua formed the same clade with *P. latirostris*, supported by bootstrap 71% NJ.

These results uncovered that all samples from different parts of Indonesia were divided into five different species of catfish based on the mtDNA Cyt B sequence. The five different species belonged to three different families: Bagridae, Pangasiidae, and Ariidae. Our samples generally consisted of Bagridae family that was distributed from Sumatera, Central Java, and Kalimantan. Interestingly, we found that samples from Bojonegoro, East Java belonged to Pangasiidae. The phylogenetically distinct Papuan samples had their places in the Ariidae, which mostly contained the coastal to estuaries catfish [28], while our samples were collected from the river sources.

These results of our analyses were identical to a previous study using the COX-III sequences [4]. Besides, these results also supplemented the research done by Dodson and Lecomte [27], which determined that Indonesian catfish were comprised of three species based on the mtDNA Cyt B: *Hemibagrus planiceps* from Java; *Hemibagrus bongan* from Kalimantan, and *Hemibagrus velox* from Sumatra. In fact, there were additional catfish species from different parts of Indonesia discovered in this study, despite the similarity of their morphological features. The geographical adjustments which have occurred in Indonesian lands throughout the geological history have influenced the speciation and the alterations of the mtDNA of Indonesian catfish species, forming their recent common lineages. Therefore, to distinguish the group of Indonesian catfish, the phylogenetic analysis based on the DNA sequence is definitely needed for the species’ successful conservation.

## Conclusion

Indonesian catfish were divided into five clades or monophyletic groups based on Cyt B gene. Catfish sample KR and MP (Sumatra); MS and BB (Kalimantan); and PM (Java) were clustered with *H. nemurus* and *H. wyckioides* (Bagridae family). Samples from Kalimantan (KS) and one sample of KR (KR1) from Sumatra were clustered with *S. seenghala* and *H. spilopterus* (Bagridae family). Other catfish samples from Java (BSBJ) were clustered with *P. pleurotaenia* (Pangasiidae family), while samples EM were together with *M. cavasius* (Bagridae family). Finally, catfish samples from West Papua were clustered with *P. latirostris* (Ariidae family).

## Authors’ Contributions

RW designed and collected samples for this study. DVM, HAN, ZPA, and YDRBRS carried out the research in the laboratory. RW, DVM, and HAN analyzed, wrote, and revised the manuscript. All authors have read and approved the final manuscript.
